# The interplay between host-specificity and habitat-filtering influences sea cucumber microbiota across an environmental gradient of pollution

**DOI:** 10.1186/s40793-024-00620-2

**Published:** 2024-10-13

**Authors:** Sheena Suet-Wah Chung, Khan Cheung, Bovern Suchart Arromrak, Zhenzhen Li, Cham Man Tse, Juan Diego Gaitán-Espitia

**Affiliations:** 1 The Swire Institute of Marine Science and School of Biological Sciences, The University of Hong Kong, Pok Fu Lam, Hong Kong SAR China; 2 GEOMAR Helmholtz Centre for Ocean Research Kiel, Kiel, Germany; 3 Jiangsu Key Laboratory of Marine Bioresources and Environment and Jiangsu Key Laboratory of Marine Biotechnology, Jiangsu Ocean University, Lianyungang, China; 4 Institute for Climate and Carbon Neutrality, The University of Hong Kong, Hong Kong SAR, China

**Keywords:** Microbial community, Holothurian, Nutrient pollution, Environmental gradient, Host-microbiome

## Abstract

Environmental gradients can influence morpho-physiological and life-history differences in natural populations. It is unclear, however, to what extent such gradients can also modulate phenotypic differences in other organismal characteristics such as the structure and function of host-associated microbial communities. In this work, we addressed this question by assessing intra-specific variation in the diversity, structure and function of environmental-associated (sediment and water) and animal-associated (skin and gut) microbiota along an environmental gradient of pollution in one of the most urbanized coastal areas in the world. Using the tropical sea cucumber *Holothuria leucospilota*, we tested the interplay between deterministic (e.g., environmental/host filtering) and stochastic (e.g., random microbial dispersal) processes underpinning host-microbiome interactions and microbial assemblages. Overall, our results indicate that microbial communities are complex and vary in structure and function between the environment and the animal hosts. However, these differences are modulated by the level of pollution across the gradient with marked clines in alpha and beta diversity. Yet, such clines and overall differences showed opposite directions when comparing environmental- and animal-associated microbial communities. In the sea cucumbers, intrinsic characteristics (e.g., body compartments, biochemistry composition, immune systems), may underpin the observed intra-individual differences in the associated microbiomes, and their divergence from the environmental source. Such regulation favours specific microbial functional pathways that may play an important role in the survival and physiology of the animal host, particularly in high polluted areas. These findings suggest that the interplay between both, environmental and host filtering underpins microbial community assembly in *H. leucospilota* along the pollution gradient in Hong Kong.

**Supplementary Information:**

The online version contains supplementary material available at 10.1186/s40793-024-00620-2.

## Introduction

Metazoans harbour diverse and dynamic microbial communities (microbiome) that play essential roles in the ecology and function of their animal host [1]. Symbiotic microorganisms influence host metabolic processes [29, 49, 50], development [12], immune responses [21, 63], reproduction [7, 95], behaviour [96] and survival [59]. However, microbial communities and the interactions with their hosts are not static and can vary across time (e.g., host’s development or seasonality, [59, 97]) and space (e.g., host’s body plan or geography, [47, 76]). Thus, to understand the ecological dynamics of animal hosts in a changing world, it is becoming more important to understand the mechanisms and drivers underpinning the origin and regulation (functional and structural) of their associated microbial communities [62].

Host-associated microbiomes are shaped by a diversity of evolutionary and ecological processes that can be explored through the framework used in community ecology. Under this framework, the formation of microbial communities is seen as the outcome of selective processes in which a larger species pool is subjected to a set of biotic and abiotic filters [51]. For instance, habitat/environmental filtering is usually considered one of the dominant forces in structuring communities consisting of habitat-specialized species [13]. In this case, the habitat/environment is part of the selective processes as it plays a dual role acting as a source for the hosts’ microbiome and also influencing the composition and dynamics of the established microbial community [57]. As a result, host intra- and interspecific variation in microbiome composition is expected to be lower for organisms inhabiting similar environments in comparison to their counterparts living in more divergent conditions [57]. Hosts, however, can also influence associated microbiomes in several ways, such as through selective feeding or filtering [40, 102]. This may result from evolutionary processes that produce patterns in which closely related host species harbour similar microbiota even if they inhabit different environments [93, 94]. In any case, environmental and host filtering are aligned with the fundamental idea that deterministic processes dictate the microbial community assembly in natural populations [80, 105, 111, 116].

Previous studies have also suggested that stochastic processes can influence microbial community assembly [84, 127]. These processes include random dispersal potential and colonization chance of microbes [128], random arrival sequence to host from the environment during dispersal [8], changes in community abundance and survival due to random speciation, extinction and ecological drift [14]. Under this paradigm, species present in the microbial community would be independent and unpredictable, with no specific occurrence pattern or relation with the respective niche [92]. This provides us with a wider perspective when studying processes underpinning microbial community assembly. What is worth mentioning is that deterministic and stochastic processes are not mutually exclusive [6]. Both processes work together in structuring microbial communities although a stronger contribution of a particular process during the assembly may occur depending on the investigated model and ecosystem [116].

In marine environments, the interplay between deterministic and stochastic factors is an important driver of inter- and intra-specific differences in organismal characteristics across heterogeneous seascapes. In these systems, gradients of environmental conditions are known to influence geographic variation in attributes such as physiology [37–39], life-history [88], zonation [72], behaviour [19], intra- and inter-specific genetic diversity [126], stress tolerance and phenotypic plasticity [37, 39] in natural populations. As such, it would be expected that environmental gradients would also influence variation and phenotypic differences in other organismal characteristics, including the structure and function of host-associated microbial communities. In fact, gradients in sea surface temperature and salinity are known to influence symbiotic microbial assemblages in benthic marine species [11, 60, 85] and coastal ecosystems [5, 109, 121, 122]. While these natural environmental gradients may have a fundamental role in modulating ecological patterns of marine microbiomes, anthropogenic-mediated gradients (e.g., nutrient pollution) can potentially induce drastic changes in these patterns by altering ecological dynamics, the origin, and regulation of host-associated microbial communities [22, 78, 98, 129].

In this study, we aimed to assess to what extent deterministic and/or stochastic processes along an anthropogenic-mediated gradient of pollution (nitrogen/phosphorus) influence intra-specific variation in diversity, structure, and function of host-associated microbial communities in marine organisms. Here, our model system was *Holothuria leucospilota,* a tropical sea cucumber that dominates shallow waters in Hong Kong and the Indo-Pacific region. *H. leucospilota* is a deposit-feeding species that assimilates organic matter from surface sediments (including bacteria, benthic phytoplankton, meiofauna, and organic detritus), which serves as a constant environmental source for microbe acquisition [40, 86]. However, *H. leucospilota* is characterised by the capacity to secrete secondary metabolites from the skin, gonads, and guts, which have antibacterial and antifouling properties [20], potentially allowing them to regulate the influence of environmental microbial reservoirs. Based on 16S amplicon sequencing, we examined both sea cucumber (skin and intestine) and environment (sediment and water) microbiomes along a pollution gradient in Hong Kong, one of the busiest ports and highly urbanized areas in the world. If environmental filtering is the main driver of microbial community assemblies along the pollution gradient, then similar host and environmental microbiomes will be observed within sites whilst higher microbiome variation would be expected across the gradient. In this context, microbial dispersal patterns and ecological drift might also play an important role in shaping inter-individual microbiome variation within sites [99]. Conversely, if host filtering and specificity are the main drivers of microbial community assembly in *H. leucospilota*, then low inter-individual variation in sea cucumber microbiome composition would be expected along the pollution gradient independently of the variation in the environmental microbial communities. However, intra-individual variation would be expected as sea cucumber skin and guts have different antibacterial properties [20].

## Materials and methods

### Sampling sites and environmental data

**Fig. 1:**
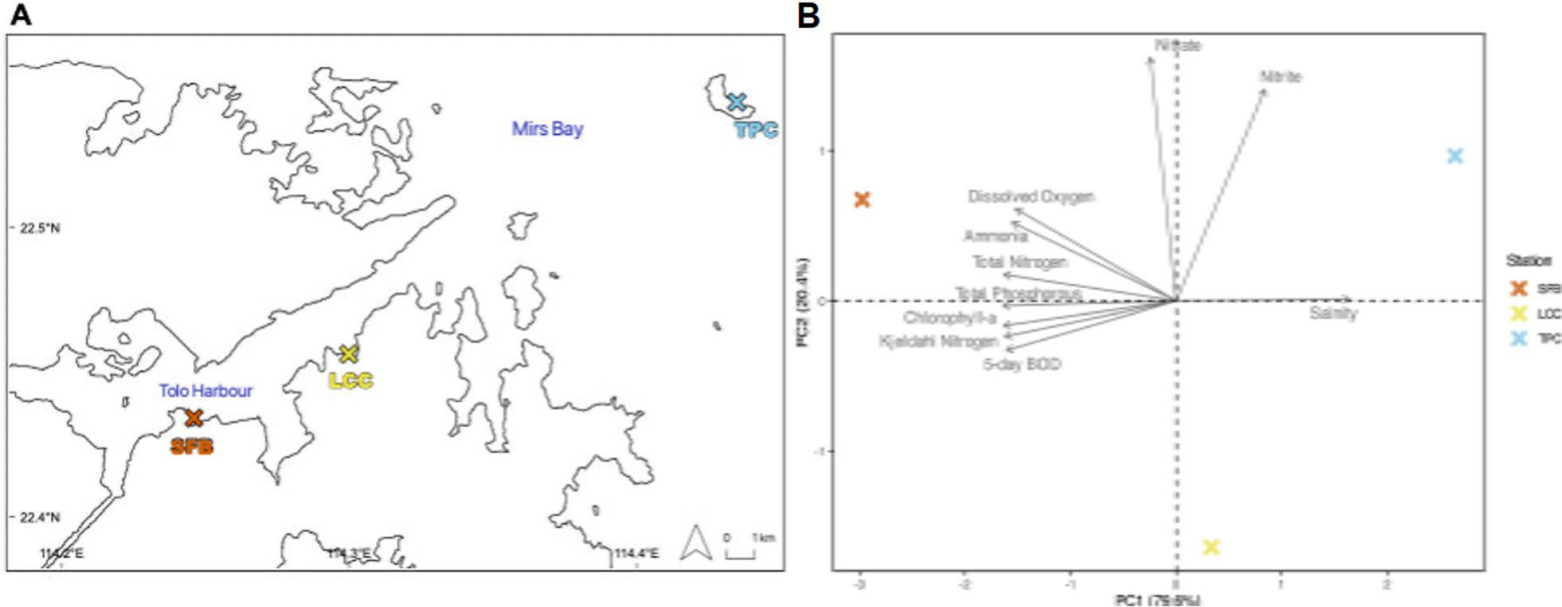
Sampling sites and water stations (**A**). PCA analysis for eight environmental parameters in the respective water station of each site (**B**)

In Hong Kong, the Tolo Harbour (TH; 22°43' N, 114° 22' W) is characterised by a gradient of chemical pollution that has profound implications for the diversity and distribution of benthic organisms, from corals [28] to microbial communities in the sediments [15, 16]. For our experimental design, we first assessed environmental parameters along the pollution gradient in the Channel Water Control Zone of TH. This information was obtained from the marine water quality database of the Hong Kong Environmental Protection Department (EPD) (available from: https://cd.epic.epd.gov.hk/EPICRIVER/marine/). Parameters such as salinity, total phosphorus, total nitrogen, total Kjeldahl nitrogen, nitrite, nitrate, ammonia, chlorophyll-a, dissolved oxygen, and 5-day Biological Oxygen Demand (BOD) on the surface water across ten years (2010–2020) were used for comparisons. Based on this information, we established three experimental localities, including Starfish Bay (SFB) (22° 26′ 3.0″ N, 114° 14′ 45.6″ E), Lai Chi Chong (LCC) (22° 27′ 22.4″ N, 114° 17′ 59.4″ E) and Tung Ping Chau (TPC) (22° 32′ 34.1″ N, 114° 26′ 6.5″ E) (Fig. 1). A principal component analysis (PCA) was conducted to visualize the differentiation of water quality between the experimental sites along the pollution gradient in TH.

### Animals and sample collection

Samples from adult sea cucumbers *Holothuria leucospilota* and their surrounding environments (water and sediments) were collected in December 2019 from each of the field sites. Sea cucumbers (5 animals per site, separated at least by 10m) were isolated in Ziploc bags filled with water from the collection site. In addition, surface sediment samples of the top 3 cm (n = 3 per site) were collected around the sampled animals using sterile 15 mL falcon tubes. In parallel, 2L seawater samples (n = 3 per site) were also collected using sterile plastic bottles wrapped with aluminium foil. All samples were immediately stored in ice for transportation and processed immediately in the laboratory at the University of Hong Kong (HKU).

At HKU, sea cucumbers were sacrificed by sectioning their anterior part close to the oral nerve ring, which accounts for the principal nervous component (No ethical approval is required at HKU for research performed on invertebrates). Then, animals were dissected to obtain microbial samples from the body surface and guts. For this, sea cucumbers were rinsed under sterile seawater twice to remove particulates such as sand, algae and other loose organic matter loosely attached to the surface. Then, a sterile cotton swab was rolled over the whole outer body surface to collect microbial samples from the skin. For the gut microbiome collection, the outer surface of the sea cucumbers was first sterilized with 70% ethanol to reduce contamination. After that, the ventral part of the animal was cut open with a sterile scalpel. A 2 cm segment of the midgut including the luminal epithelium was then collected from each animal. All skin and gut samples were individually placed into sterile 1.5 mL microcentrifuge tubes, snap-frozen in liquid nitrogen, and stored at − 80 ℃ until required for further analysis. For the seawater samples, a two-step filtering process was conducted. First, samples were passed through Millipore membrane filters of 0.45µm pore size to remove large particles, and water was then filtered again using Millipore membrane filters of 0.22 µm pore size. These last filters were transferred to 15 mL falcon tubes. Sediment samples were centrifuged (4000 g × 5 min), and the supernatant of excess seawater was removed without disturbing the sediment. Sediment and water samples were stored at − 80 ℃ until required for further analysis.

### DNA extraction and 16s rRNA amplicon sequencing

DNA from sediment and sea cucumber (skin and gut) samples was extracted using the DNeasy PowerLyzer PowerSoil Kit (Qiagen, Germantown, MD). DNA from seawater samples was extracted using the DNeasy PowerWater Kit (Qiagen, Germantown, MD), following the manufacturer’s instructions. In both cases, the same batch of DNA extraction kits was used for all the samples. Overall, we followed guidelines for sequence-based analyses of microbial communities [30] to avoid any potential contamination of samples originating from reagents or the laboratory environment. As part of this, DNA extractions were performed in the Marine Molecular Lab (HKU) under a sterilised laminar flow hood. After extractions, DNA concentrations were verified using BioDrop (Biochrom, UK), and DNA quality was checked via agarose gel electrophoresis. Total genomic data was submitted to Novogene Bioinformatics Technology Co., Ltd., Beijing, China for amplicon sequencing. The V3-V4 hypervariable region of the 16S ribosomal RNA gene was amplified with the primers 341F (5’-CCTACGGGNGGCWGCAG-3’) and 806R (5’-GGACTACNNGGGTATCTAAT-3’) [119]. PCR reactions were carried out with Phusion® High-Fidelity PCR Master Mix (New England Biolabs, US) following the manufacturer’s instructions. Amplicons from different samples were mixed in equidensity ratios and purified with the Qiagen Gel Extraction Kit (Qiagen, Germany). NEBNext® Ultra™ DNA Library Prep Kit for Illumina® (New England Biolabs, US) was used to construct DNA libraries (paired-end), following the manufacturer’s recommendations. Index codes were added, and the library quality was assessed on the Qubit® 2.0 Fluorometer (Thermo Scientific, US) and 2100 Bioanalyzer system (Agilent, US). Amplicons from different samples were mixed in equimolar amounts and sequenced on the Illumina NovaSeq 6000 platform with sequencing strategy PE250. Two negative controls were included during library preparation to check potential contamination. However, these controls were not sequenced as they did not generate any product during PCR amplification. Primary processing was done by removing barcodes, adaptor sequences and indices.

### Sequence and data processing

Raw sequence data from seawater, sediments, skin, and intestinal microbiota were processed with QIIME2 version 2021.11. After visualizing interactive quality plots and checking the reads’ quality, the DADA2 [10] pipeline was applied to demultiplex and merge pair-end reads. Quality control was performed by DADA2 on trimming, sequence error elimination, detection, and removal of chimaeras following these parameters: -p-trim-left-f 8 -p-trunc-len-f 225 -p-trunc-len-r 213. Then, a naïve Bayes classifier was trained following the RESCRIPt pipeline (Robeson II et al., 2020) using the non-redundant SSU reference dataset at 99% identity of the full SILVA 138 release [91] on the specific 16S rRNA V3-V4 amplicon region with the pair of primers stated above. Amplicon sequence variants (ASVs) classified as mitochondria, chloroplasts, archaea, eukaryotes, and unassigned taxon were subsequently excluded. Singletons and ASVs with less than 10 reads across all samples were removed (control of spurious artefacts of the PCR amplification process and/or potential sequencing errors). Samples were then centered log-ratio (clr) transformed to retain the compositional nature of microbiome datasets [44] for further downstream analysis. Raw sequences have been deposited at the National Centre for Biotechnology Information (NCBI) under the project accession number PRJNA731335.

### Statistical analysis

Statistical analysis was conducted on the ASV level of the *clr*-transformed 16S rRNA dataset. Prokaryotic community profiles were constructed at the phylum, family and genus level. The relative abundance of major phyla (> 0.1% of the total microbial community) in environmental samples was compared between sites. Then, the top ten most abundant families of the entire dataset were compared between sources and between sites by Analysis of Compositions of Microbiomes with Bias Correction (ANCOM-BC) [70]. Alpha diversity metrics including the abundance-based coverage estimator (ACE), diversity (Shannon diversity index), and evenness (Inverse Simpson’s diversity index) were computed. A comparison between each of the alpha diversity indices by sites and source was performed by two-way ANOVA followed by a post-hoc Tukey honestly significant difference (Tukey HSD) test after ensuring data normality and homogeneity with tests as above.

To address the community variation between samples collected from different sites and sources, we adopted a permutational multivariate analysis of variance (PERMANOVA) analysis and Analysis of Similarity (ANOSIM) with 999 permutations to compare their compositions. A distance matrix based on the ASVs was first constructed with the *distance* function in the “vegan” R package [83] with the Euclidean distance [44]. Then, the Adonis method via the *adonis* function with 999 permutations was used for the comparison of communities. Pairwise PERMANOVA was carried out with *pairwise.adonis* with the “pairwiseAdonis” package [2]. Permutation tests for homogeneity of multivariate dispersions (PERMDISP) were conducted using *betadisper* and *permutest* to verify significant PERMANOVA outcomes. Principal Component Analysis (PCA) was used to visualize the beta diversity matrix in the “phyloseq” package [77].

### Core analysis, differentially abundant taxa and functional prediction

Shared ASVs among sources of each site were visualized with the “MicEco” package. Core communities were defined to facilitate the interpretation of host and environmental microbiota. ASVs being present in at least 70% of samples were considered as core and rare ASVs were those that were present in fewer than 30% of samples [4]. All other ASVs were considered transient. Indicator ASVs were identified with the *multipatt* function with the “indicspecies” package [9]. Differentially abundant ASVs between sources were also identified with the ANOVA-Like Differential Gene Expression Analysis (ALDEx2) with the “ALDEx2” package [33].

Phylogenetic Investigation of Communities by Reconstruction of Unobserved States (PICRUSt2) was used to predict physiological and metabolic functions of the host and environment microbiota based on ASVs generated from the QIIME2 DADA2 pipeline [27, 65]. This procedure predicts the relative abundance of functional genes (expressed as Kegg Orthologs–KOs) in a 16S ASV community from the phylogenetic conservation of these genes in all currently sequenced and assembled prokaryotic genomes. Quality control was implemented by computing weighted nearest sequenced taxon index (NSTI) values of each ASV. NSTI evaluates the prediction accuracy of PICRUSt because it reflects the average genetic distance (measured as number of substitutions per site) between each ASV against a reference genome [27, 65]. NSTI values higher than 2 were eliminated following the developer’s guidelines [27]. PERMANOVA with 999 permutations was adopted to compare functional pathways between sources and sites. Potential differentially abundant functional MetaCyc pathways between sources were analysed by ALDEx2. Those that were significantly differentially abundant (*p* < 0.01) were then visualized with the “ComplexHeatmap” package [48]. All R packages mentioned were implemented in RStudio ver. 1.2.5019. In order to support and facilitate scientific reproducibility, all analyses performed were included in the script as part of the supplementary materials.

## Results

### Environmental gradient based on water quality

The averaged environmental parameters in the PCA analysis reflected a water quality trend from TM6 (SFB) to MM5 (TPC) (Fig. 1, Table S1). The seven environmental parameters varied between sites. Nitrogen (nitrite and nitrate) and salinity have a greater contribution to the variation in TPC whilst ammonia and phosphorus to LCC. Overall, PC1 explained most of the variation observed (79.6%) among sites and this was consistent with the geographic distribution, supporting the occurrence of a pollution gradient along Tolo Harbour (Fig. 1). Based on the environmental data, sites were categorized into three relative pollution levels along the Tolo Harbour Channel (SFB as highly polluted, LCC as moderately polluted, and TPC as low polluted site).

### Sequencing results

A total of 2,117,314 sequences were obtained after quality control from all 48 samples (sediment, seawater, sea cucumber skin, and guts) from each of the three sites (SFB, LCC, and TPC) along the pollution gradient. The effective reads ranged from 14,872 to 76,342 with an average of 44,110 (± 17,337 SD) reads per sample. The maximum total number of ASVs generated was approximately 300 (Fig. S1) after filtering unwanted taxa and annotating sequences to the genus level.

### Microbial community composition in environmental and host samples

**Fig. 2:**
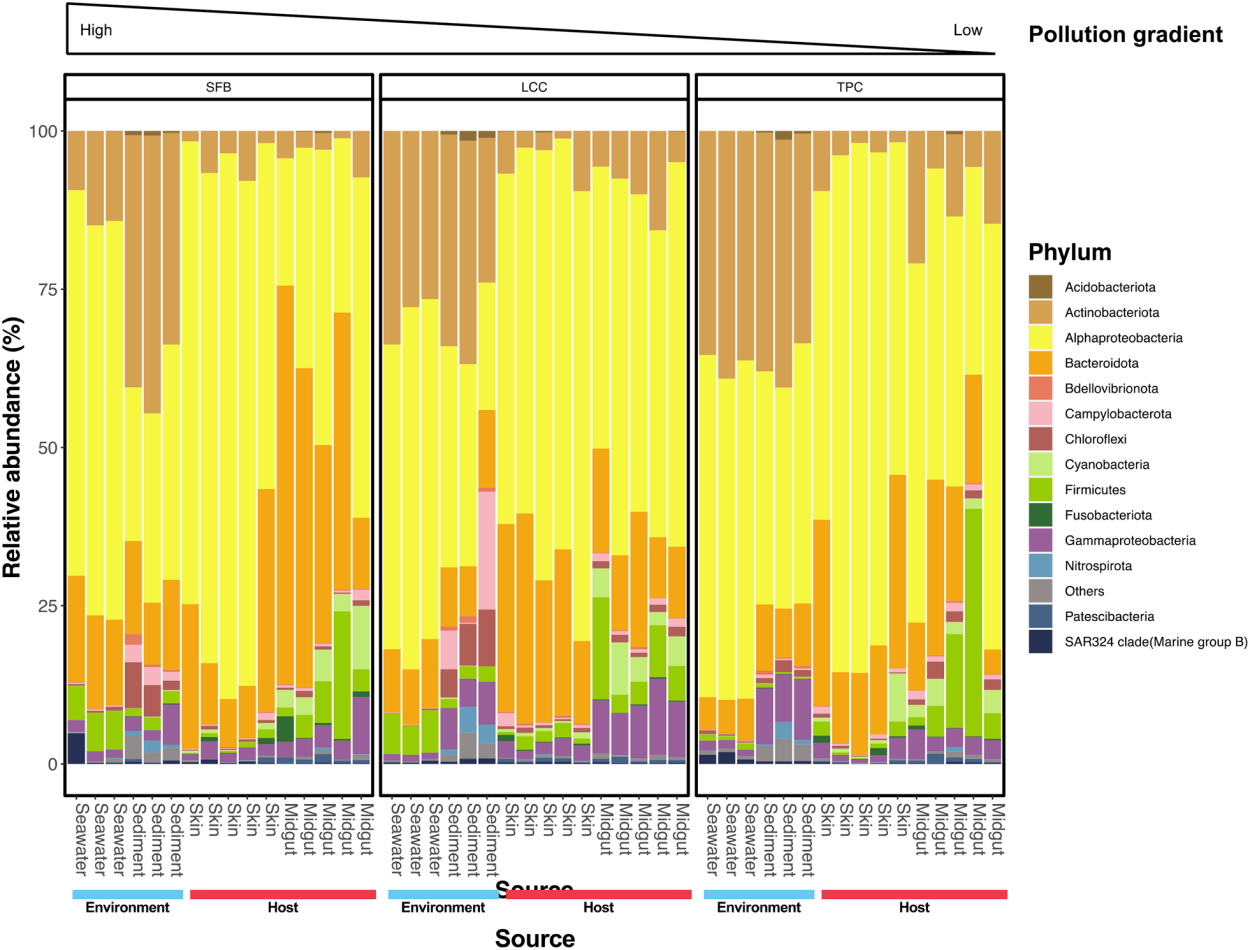
Taxonomic plot showing the community composition of the major phyla (class for Proteobacteria), which are those present in more than 0.1% of the total microbial community. “Others” denote phyla of less than 0.1% of the total microbial community

**Fig. 3:**
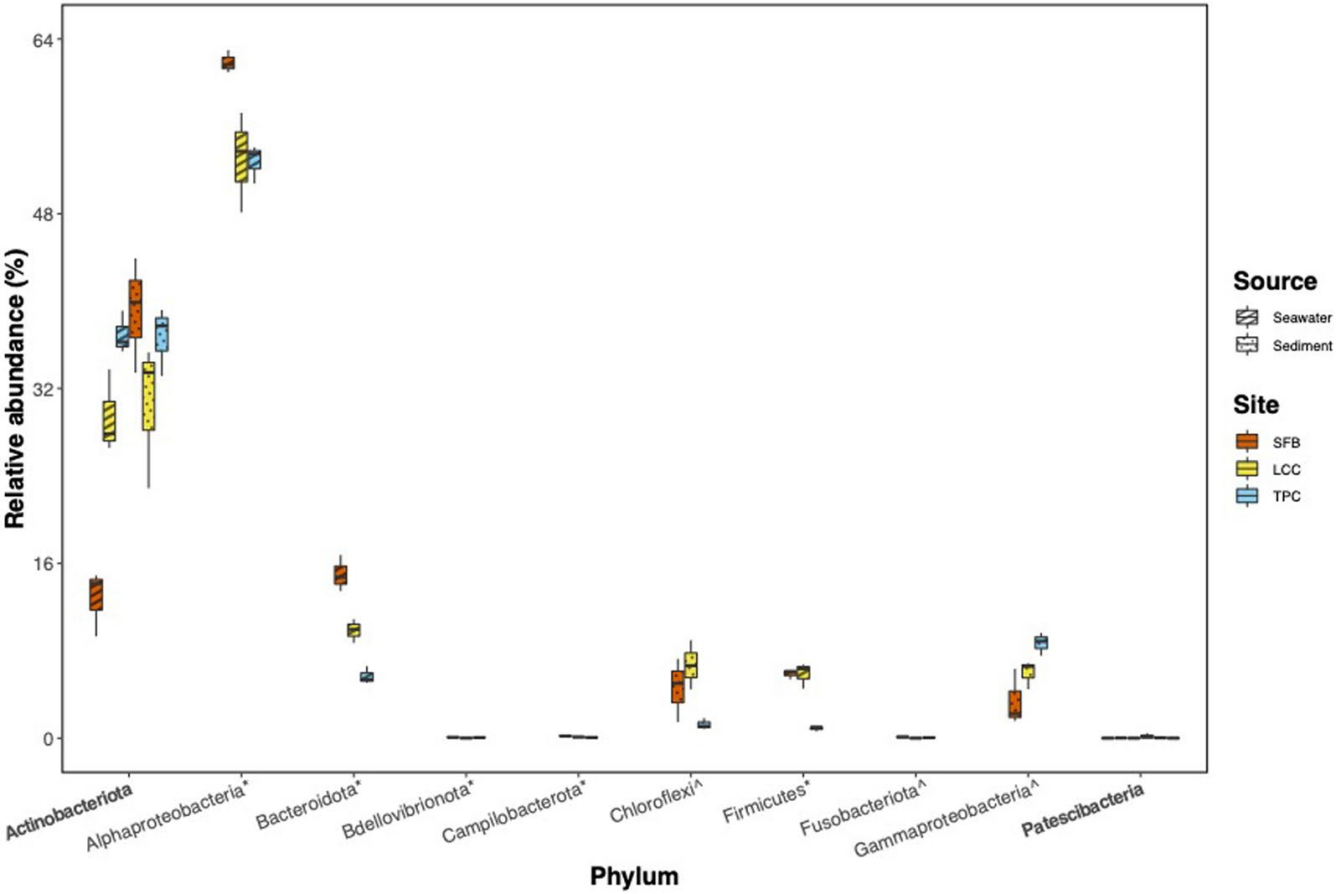
Phyla that are statistically significant between sites in sediment (asterisk annotation), seawater (caret annotation) or both (in bold) samples

The taxonomic composition of microbial community abundance on the phylum level of environmental samples showed *Pseudomonadota* (Class *Alphaproteobacteria,* 56.7%), *Actinobacteriota* (23.6%), *Bacteroidota* (11.4%), *Firmicutes* (4.9%), and *Gammaproteobacteria* (1.3%), as the most abundant phyla in seawater. For sediments, *Actinobacteriota* (35.6%), *Alphaproteobacteria* (32.9%), *Bacteroidota* (10.9%), *Gammaproteobacteria* (6.0%), and *Chloroflexi* (4.0%) were the main groups (Table S2). However, the microbial composition varied between sites, with more accentuated differences in the contribution of major phyla (> 0.1% of the total microbial community) in more polluted sites. These differences in microbial composition between seawater and sediments gradually declined in moderated and less polluted sites, particularly for *Alphaproteobacteria* and *Actinobacteriota* (Figs. 2 and 3, Table S3), albeit some site-specific and source-specific departures of the trend. In the moderately polluted site, *Campilobacterota* and *Chloroflexi* increased their overall contribution to the sediment microbiota while remaining invariable in the seawater (Fig. 2). In the sea cucumber host, the microbiota was also dominated by *Alphaproteobacteria*, *Bacteroidota*, *Actinobacteriota*, *Firmicutes,* and *Gammaproteobacteria* (Table S2). This community composition differed from the local environment and varied along the cline (Fig. 2), with a major contribution of the family *Rhodobacteraceae* (*Alphaproteobacteria*; Figs. S2 and 4) and species from the associated genera *Ruegeria*, *Dinoroseobacter,* and *Oceanicella* (Fig. S3).

**Fig. 4:**
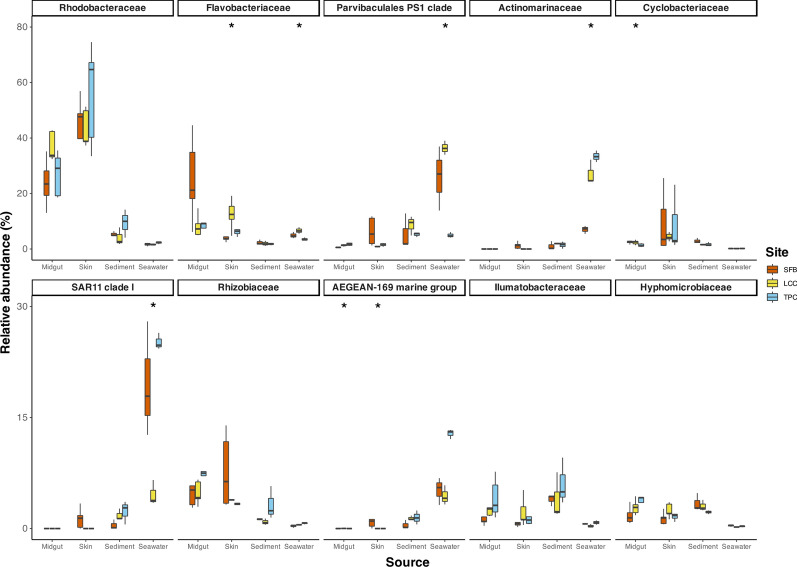
Barplot showing the comparison of the relative abundance of the top ten most abundant family taxa across sites for each sample source. Families that are statistically significant between sites in each source are marked with an asterisk (See Table S5 for test statistics details)

Taxonomic analysis revealed an overall dominance of 10 families in host-associate tissues, with a major contribution of *Rhodobacteraceae* followed by *Flavobacteriaceae, Parvibaculales PS1 clade, Actinomarinaceae, Cyclobacteraceae, SAR11 clade 11, Rhizobiaceae, AEGEAN-169 marine group, Ilumatobacteraceae*, and *Hyphomicrobiaceae* (Fig. 4). Families showing statistically significant differences in the relative abundance were associated with the site locations and sources (Table S4 and Table S5). For example, Rhodobacteraceae relative abundance was clearly different between host-associated and environmental samples. However, no significant differences were detected across sites from their corresponding sample sources (Fig. 4 and Table S4). Some families showed a clinal difference in their relative abundance across sites (e.g., *Actinomarinaceae* from seawater samples) (Fig. 4 and Table S5).

### Community structure along the pollution gradient

**Fig. 5:**
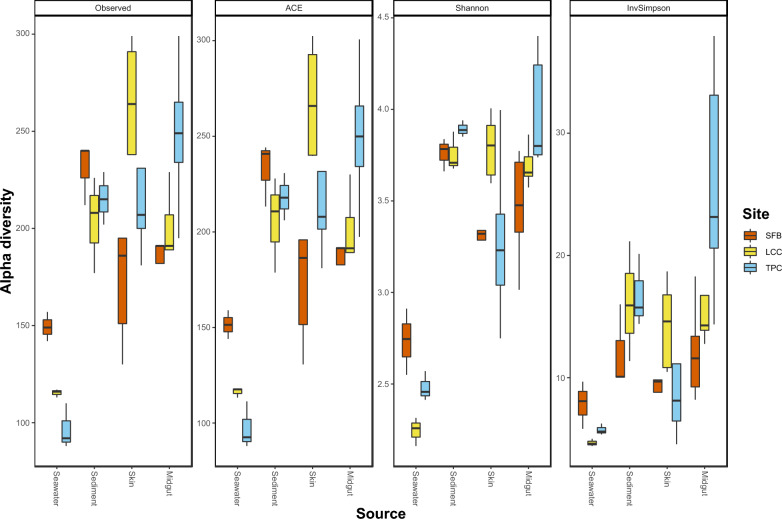
Alpha diversity metrics boxplot of sites by sources

A comparison of alpha diversity indices revealed statistically significant differences between samples from skin and gut, as well as between sites (Table 1, Table S6). Significant interaction was detected between these two experimental factors. Seawater microbiota evidenced lower species richness and diversity, while the other three sources shared a similar richness and diversity regardless of sites (Fig. S4). Overall, environmental samples from the highly polluted site (SFB) were higher in richness and diversity than the two other sites (Fig. 5). For the moderately polluted site (LCC), a higher richness and diversity were observed in skin samples. Midgut microbiota is the richest and most diverse community in samples from the low polluted site (TPC).

**Table 1:** Two-way ANOVA of alpha diversity indices of microbial communities between different sites and sources

Groups	df	ACE		Inverse Simpson		Shannon	
		*F*	*p*-value	*F*	*p*-value	*F*	*p*-value
Source	3	14.84	**< 0.0001**	3.78	**0.019**	30.84	**< 0.0001**
Site	2	5.43	**0.0087**	11.31	**0.00015**	5.83	**< 0.0064**
Source × Site	6	5.19	**0.00062**	3.18	**0.013**	4.89	**< 0.00096**

*p*-values in bold denote statistical significance at *p* < 0.05

*ACE* abundance-based coverage estimator, *Inverse Simpson* inverse Simpson’s diversity index, *Shannon*: Shannon diversity index

**Fig. 6:**
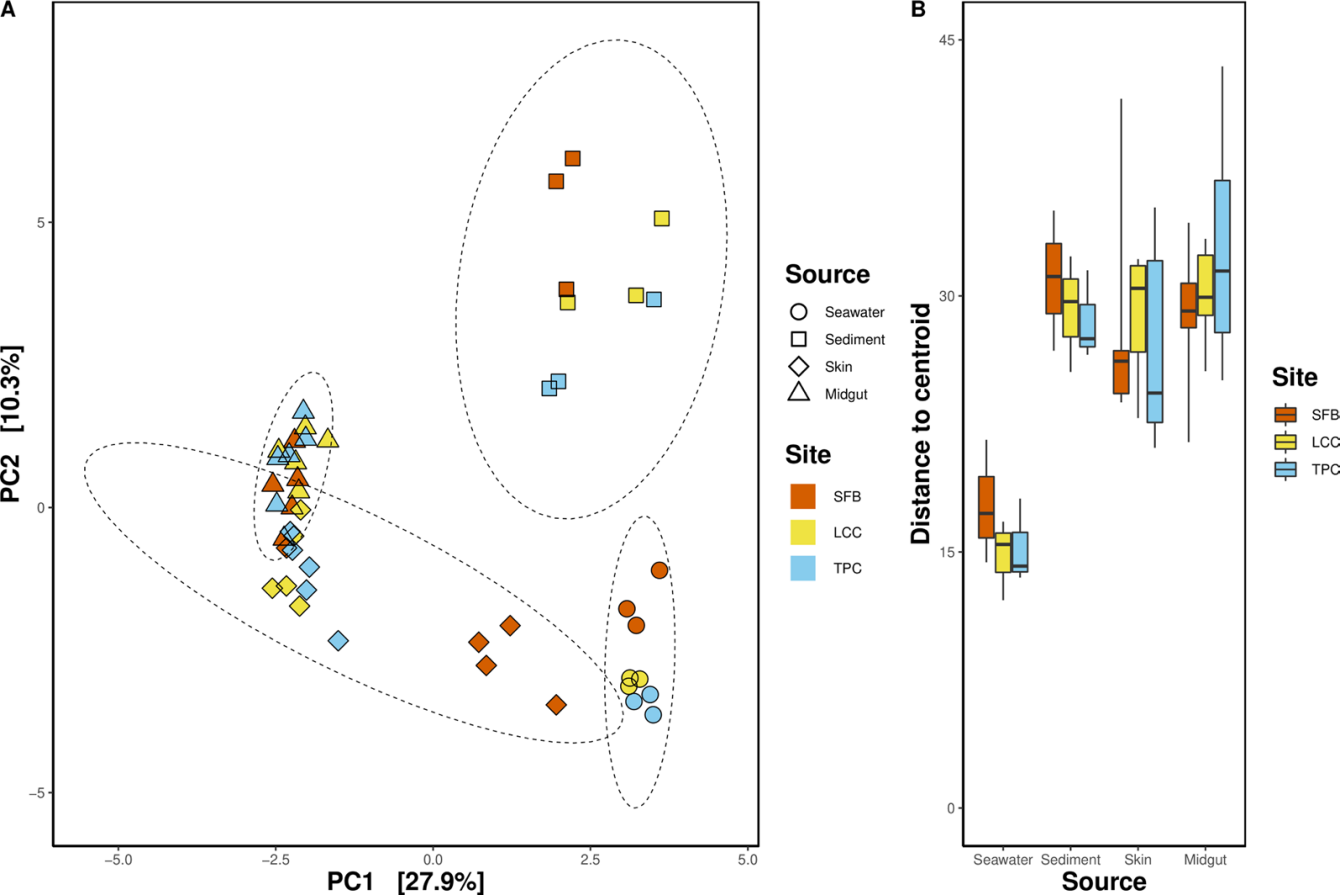
PCA plot (**A**) by Euclidean distance, eclipses in dashed lines denote the multivariate 95% confidence interval by source. Boxplot of distance to centroid (**B**)

Overall, differences in microbial community structure were observed between seawater, sediment, skin, and midgut samples collected from different sites. Microbial communities from different sources were all distinct from one another (Table 2), although skin and midgut samples clustered closer together (Fig. 6A). However, these similarities between skin and midgut microbiota composition varied across sites (Table 3). A deviation of seawater microbiota in Starfish Bay could be observed along PC2, away from the two other sites (Fig. 6A). Community structure showed a high influence on the interaction between source and site factors as can be seen in the clustering tendency of the samples (Fig. 6A). A general higher to lower dispersion trend in beta diversity from highly polluted to low polluted sites was observed in seawater and sediment samples. On the other hand, dispersion patterns in hosts across the three sites did not align with that of the environmental sources. In midgut samples, a general trend of higher beta diversity dispersion in the least polluted site was observed (Fig. 6B, Table 3).

**Table 2:** Comparison of beta diversity of microbial communities at the amplicon sequence variant (ASV) level between different sites and sources

Groups		PERMANOVA		ANOSIM		PERMDISP	
	df	*F*	*p*-value	*R*	*p*-value	*F*	*p*-value
Site	2	1.71	**0.038**	− 0.003	0.443	0.383	0.69
Source	3	10.88	**0.001**	0.75	**0.001**	20.18	**0.001**
Site × Source	6	1.85	**0.003**	–	–	–	–

*p*-values in bold denote statistical significance at *p* < 0.05

*PERMANOVA* permutational multivariate analysis of variance, *ANOSIM* analysis of similarities, *PERMDISP* test for homogeneity of multivariate dispersions

**Table 3:** Pairwise comparisons of beta diversity by source

Pairwise comparisons between source	PERMANOVA		PERMDISP
	*F*	*p*-value	*p*-value
Midgut vs. Skin	4.58	**0.001**	0.442
Midgut vs. Sediment	9.89	**0.001**	0.135
Midgut vs. Seawater	16.05	**0.001**	**0.002**
Skin vs. Sediment	8.96	**0.001**	0.691
Skin vs. Seawater	12.69	**0.001**	**0.003**
Sediment vs. Seawater	7.17	**0.001**	**0.001**

*p*-values in bold denote statistical significance at *p* < 0.05

*PERMANOVA* permutational multivariate analysis of variance, *PERMDISP* test for homogeneity of multivariate dispersions

### Environmental and sea cucumber core microbiota

**Fig. 7:**
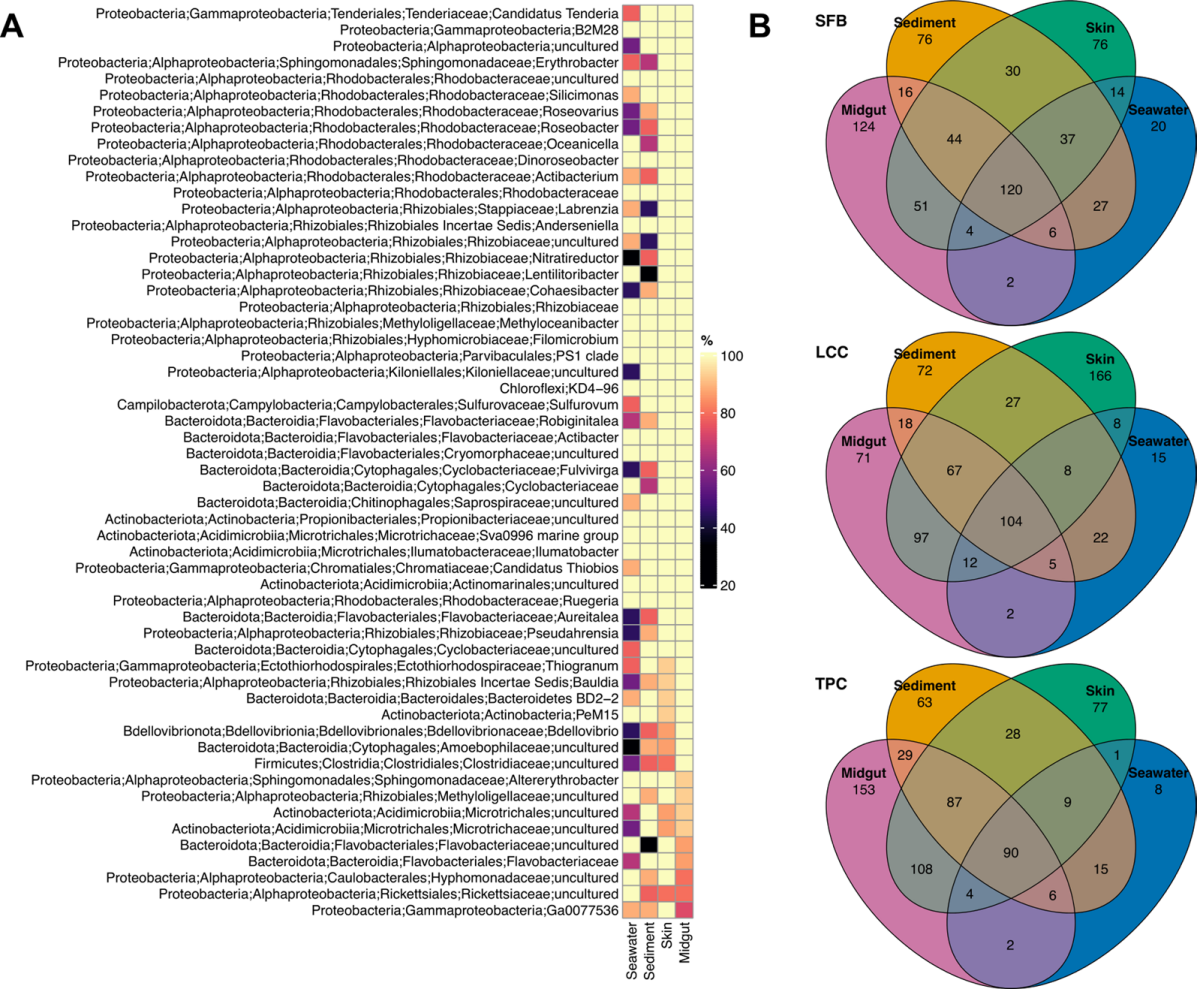
Heatmap showing the percentage of samples by source containing the core ASVs (**A**). Only core taxa that appear in at least 80% of all samples were shown. Venn diagrams of the number of shared taxa between the four sources in three sites (**B**)

A majority of ASVs in microbiota communities (on average 73.59% ± 6.74 SD) were considered rare because of their low representation among samples (present in less than 30% of samples, as defined in Bjork et al., 2018). On average, 13.64% (± 3.38 SD) of ASVs were considered as core (Table 4). Around 20.54% of the core ASVs in the midgut or skin were not found in either of the environmental samples, although a small number of core ASVs were shared among the four sources (Fig. 7). On top of that, 13 ASVs belonging to the core midgut microbiome were absent from sediment samples (Table S7). Indicator species also comprised on average 13.97% (± 9.43 SD) of the microbial community (Table S8). The contributions of unique ASVs from different sources were different between the three sites. The highest contribution of unique ASVs in Starfish Bay and Tung Ping Chau originated from midgut samples, while more unique skin ASVs were found in Lai Chi Chong (Fig. 7).

**Table 4:** ASVs belong to core, transient, rare community, and indicator species in samples of the four different sources

	Core (%)	Transient (%)	Rare (%)	Indicator (%)
*Environment*				
Seawater	8.61	8.26	83.13	8.33
Sediment	15.43	16.99	67.58	26.96
*Host*				
Skin	15.79	13.64	70.57	14.71
Midgut	14.71	12.2	73.09	5.88

### Microbial taxa and functioning profile significantly associated with host samples

**Fig. 8:**
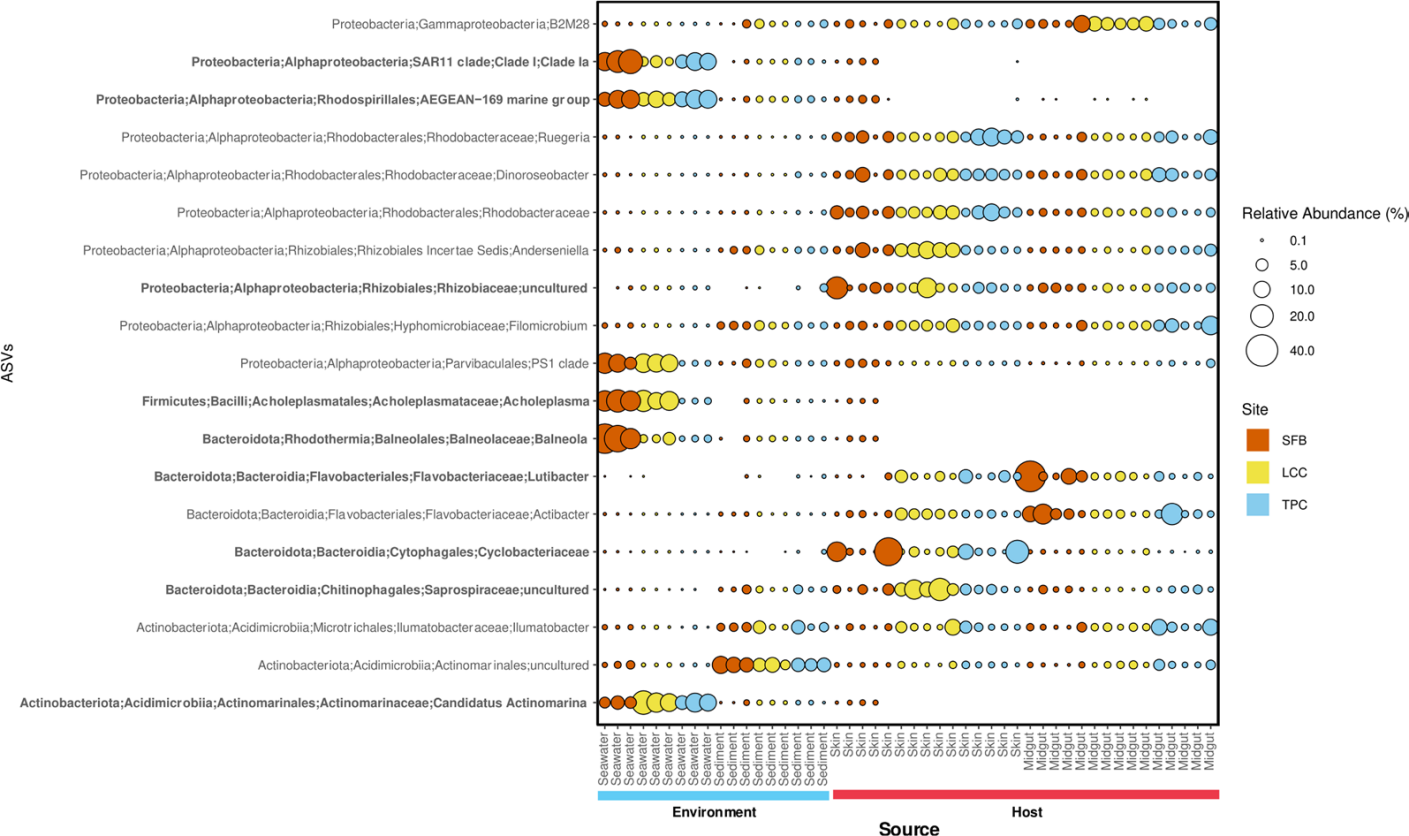
Bubble plot showing the differentially abundant taxa across sources. Only taxa with an overall relative abundance above 1% were shown. Taxa in bold are also indicator species

**Fig. 9:**
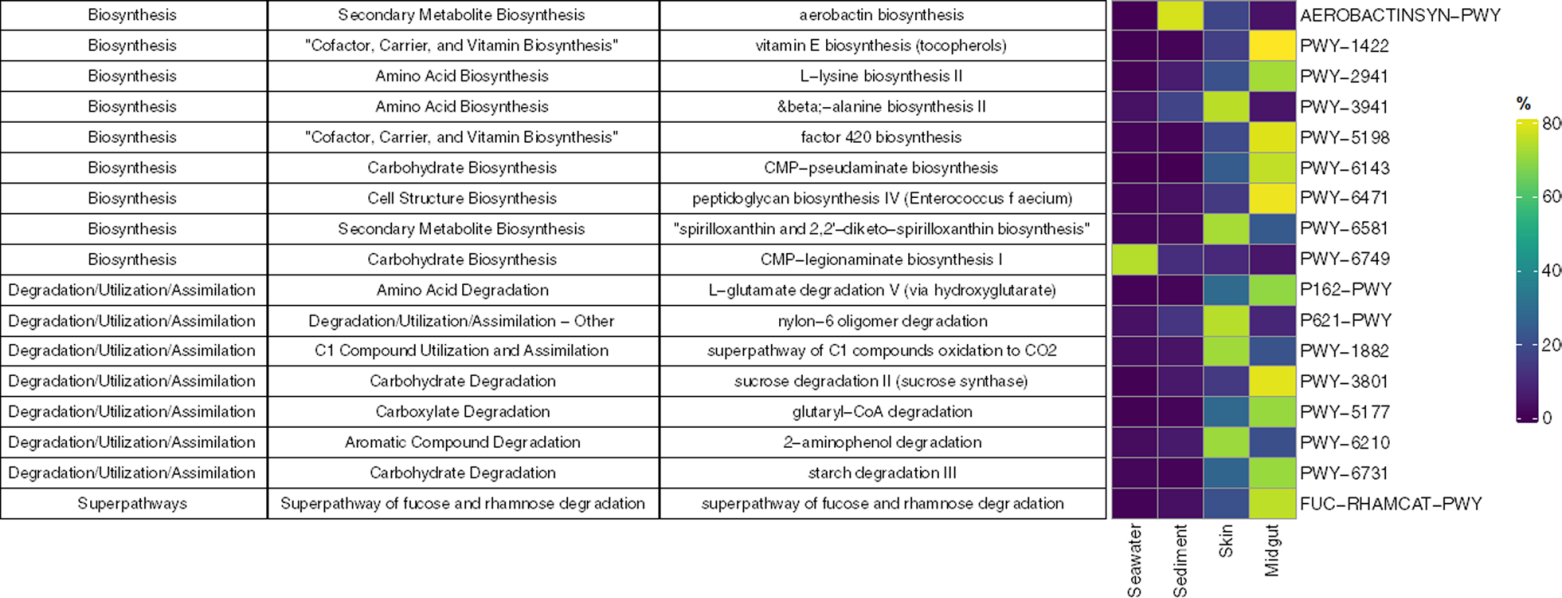
Functional pathways that are differentially abundant between sources. Only pathways with a relative abundance of over 70% in one of the sources were shown in the above

A comparison between sources in ALDEx2 analysis yielded 166 differentially abundant ASVs (Fig. 8, Table S9). A majority of ASVs were affiliated with *Rhodobacteraceae*, *Flavobacteriaceae*, *Rhizobiaceae*, *Sphingomonadaceae,* and *Cyclobacteriaceae*. 126 of the differentially abundant ASVs were also considered as bacteria indicator taxa. 334 pathways were revealed as significantly differentiated profiles. Pathways associated with the cofactor, carrier, and vitamin biosynthesis and degradation of amino acid and carbohydrate generally occurred more abundant in midgut samples. Whereas pathways involved in inorganic nutrient metabolism, amine, and polyamine degradation, and aromatic compound degradation were more likely to be found in seawater samples (Fig. 9, Table S10). Functional pathways distinguished between sources but not sites (Fig. S5, Table S11).

## Discussion

Environmental gradients can influence ecological interactions and phenotypic characteristics (e.g., physiology, microbiome) of natural populations. In this work, we investigated the extent to which an environmental gradient of pollution in one of the most urbanized coastal areas in the world, modulates the interactions of animal hosts and their associated microbial communities. By assessing intra-specific variation in the diversity, structure, and function of environmental and animal-associated microbiota in the tropical sea cucumber *Holothuria leucospilota*, we tested the interplay between deterministic (e.g., environmental/host filtering) and stochastic (e.g., random microbial dispersal) processes underpinning host-microbiome interactions and microbial assemblages. Overall, our results indicate that microbial communities are complex and vary in structure and function between the environment and the animal hosts. However, these differences can be modulated by the level of pollution across the gradient with marked clines in alpha and beta diversity. Yet, such clines and overall differences showed opposite directions when comparing environmental- and animal-associated microbial communities. These findings suggest that the interplay between both, environmental and host filtering underpins microbial community assembly in *H. leucospilota* along the pollution gradient in Hong Kong.

Excessive nutrient and metal loading, driven by rapid urban development, is a major threat to coastal and marine ecosystems worldwide, leading to profound changes in biodiversity, biochemical processes, and ecosystem functioning [28, 52, 54, 58, 74, 75, 113]. In Hong Kong waters, at a larger scale, this chemical pollution is influenced by the Pearl River [42], generating a strong west–east gradient in nitrogen (west: nitrated dominated; east: dissolved organic nitrogen dominated) and heavy metals, with levels exceeding thresholds for sediment toxicity [42, 54]. At smaller scales, other independent west–east pollution gradients can be found in some areas of Hong Kong (e.g., Tolo Harbour) influenced by the high sewage loading, the tidal hydrodynamics, and the seascape structure (Fig. 1). Both, large- and small-scale gradients of pollution in Hong Kong have been linked with recent faunal changes in benthic species [54, 82], and the alteration of the spatial distribution and loss of foundational species such as hard corals [28]. Our study revealed that environmental microbial communities are also influenced by the geographic trend in pollution that exists along the Tolo Harbour (west–east), a potential consequence of clinal differences in nutrient availability, especially for nitrogen and phosphorus. Similar correlations between microbial composition in sediments and seawater have been documented along pollution gradients in other marine regions as a function of geographic clines in phosphorus [98], heavy metals, and nitrogen availability [15, 16, 25]. In our study, the west-to-east gradient of pollution showed negative clines in the contribution of some dominant alphaproteobacterial groups such as the *PS1 Clade, Balneola*, and *Acholeplasma,* while positive trends in *AEGEAN-169, Candidatus actinomarina*, and *Ilumatobacter*. Such geographic differences could be attributed to shifts in the microbial ecological niche and some degree of local adaptation. In fact, it has been shown that microbial communities in more polluted sites can exhibit higher capacity to reduce intracellular levels of heavy metals, hydrocarbons, and other environmental contaminants compared to less polluted areas [15, 16, 23]. For instance, *Balneola* sp. is a competitive organic-degrading bacteria that is known to proliferate in coastal areas with high levels of N enrichment [115]. In our study, this group exhibited an increased abundance in the highly polluted site (SFB) and decreased in the medium–low pollution areas (LCC–TPC) of the Tolo Harbour. This trend was also observed in *Acholeplasma*, a group that typically dominates coastal areas characterized by high chemical oxygen demand driven by wastewater discharges [122]. A contrasting pattern was found for *Candidatus actinomarina*, a bacteria that has the capacity to proliferate in oligotrophic marine waters [71], thanks to physiological adaptations that facilitate efficient nutrient acquisition and processing [43, 66].

Despite the general geographic trend in microbial communities, we found micro-habitat differences in the functional profiles within the more polluted site. In the inner part of the Harbour (west), significant group dispersion was observed in environmental microbiota compared to the outer parts (east: less polluted sites). The high to low variation in bacterial community composition along the cline was particularly evident between microbial communities in seawater and sediments, a pattern that may be explained by differences in their enzymatic capabilities (broader in sediments) and strategies to access organic matter that has already been degraded during passage through the water column [101]. In seawater, the lower microbial diversity was mainly dominated by *Alphaproteobacteria* followed by *Actinobacteria* and *Bacteroidota*. However, such dominance declined with the level of pollution along the Tolo harbour. These findings are partially aligned with previous studies along a eutrophication gradient in the South China Sea [69, 123, 124]. *Actinobacteria* in particular, has been well documented as a dominant group in eutrophic environments [45, 120], in which these organisms are suggested to play a wide range of ecological functions such as the decomposition of organic matter [90]. In sediments, on the other hand, the higher diversity was dominated by *Actinobacteriota*, *Alphaproteobacteria,* and *Bacteroidota.* Of these, only *Actinobacteriota* showed major changes along the cline, together with other groups such as *Gammaproteobacteria* (copiotrophs and involved in nitrate metabolism, [53, 81]) and *Chloroflexi*. Such dominance and clinal trend in the sediments contrast with the profiles observed in the seawater, highlighting the differential ecological influence of physicochemical conditions in these environments (i.e., water column and benthos). This is particularly true for nutrient load and chemical pollution, as environmental differences in these factors are known to influence variation in microbial abundance across marine gradients [18, 56].

The sea cucumber*-*associated microbiome does not fully reflect the microbial composition of the environment along the pollution gradient. Similar to the sediments, the diversity of microbial communities in the guts and skin of the sea cucumbers was higher than in the surrounding seawater. However, contrary to the sediments, sea cucumber microbiome diversity was higher in moderated (for skin) and less polluted (for guts) sites. These clinal differences were also reflected in the overall community composition where sea cucumber microbiota was dominated by *Alphaproteobacteria*, *Bacteroidota*, *Actinobacteria*, *Firmicutes*, *Gammaproteobacteria*, and *Cyanobacteria.* While the initial three phyla also exhibited dominance in environmental samples, there were evident differences in their composition between the host and environmental samples. This pattern was more pronounced at the family level*,* where the *Rhodobacteraceae* was a dominant group associated with the host samples (with a potentially major contribution to polyhydroxybutyrate metabolism and host growth; [117]). This family was considerably reduced in the environmental samples. These findings are in line with previous studies, highlighting the role of host filtering as a “modulatory tool” shaping the microbial composition from the external microbial source pool [41, 111]. In fact, these phyla have been documented as dominant groups in the guts of diverse species of sea cucumbers [32, 86, 123, 124], suggesting a conserved filtering mechanism, likely mediated by secondary metabolites [20] or immune factors [26, 46, 107]. Such a mechanism promotes/favours specific sets of microbes with potential beneficial effects for the health and survival of the sea cucumber host [125], or that display neutral and/or transient characteristics [106]. However, other mechanisms, uncoupled from the sea cucumber host, seem to have an additional influence on the microbiome of *H. leucospilota* as can be seen by the clinal differences in some microbial groups across the pollution gradient. These mechanisms are most likely associated with the regulatory influence of environmental factors and their role in shaping microbial assemblies [24, 114]. Such influence, for instance, was observed in our study with the clinal trends (west–east pollution gradient) in the abundance of some families (e.g., *Cyclobacteraceae*, *Flavobacteraceae*) that are recognized by their higher physiological tolerances (e.g., to heavy metals) and capacities to degrade organic pollutants [100]. Overall, the distinctions observed between the environmental and host samples suggest that, although the environment has an influence on the sea cucumber microbiota, the host filtering capacity plays a major role in regulating the composition and abundance of their associated microbial communities. This filtering capacity, however, may vary along geographic ranges depending on the magnitude of the environmental gradient, in this case, the level of pollution.

Intra-individual differences in sea cucumber microbiome reflect tissue-specific control of microbial communities. Group dispersion was lower in the gut microbiome across the cline while the dispersion of the skin microbiome was higher, particularly in the more polluted site. This observation can be attributed to the extent of exposure to the external environment, where the skin directly interacts with both seawater and sediments. Due to the high variability in the environment and the direct exposure of the skin, there is a higher variation in microbial composition for the skin samples. On the contrary, the gut is an enclosed system with strong control by the host on their microbial assemblages [64, 111], resulting in lower variation. Apart from the differential dispersion between the skin and gut samples, the predominant microbial groups evidenced distinct structural and functional profiles (e.g., degradation pathways associated with the skin, while both biosynthesis and degradation pathways elevated in the gut). Such intra-individual differences in the microbiome between body parts are potentially associated with their unique characteristics (e.g., biochemistry, nutrient and oxygen content), and regulatory mechanisms (e.g., secondary metabolites, [86, 104, 108], as well as the influence of the external environmental (Sylvain et al., 2020). For example, biochemical characteristics in gastrointestinal systems in diverse metazoans, including echinoderms, are suggested to favour members of *Bacteroidota* (the second most dominant phyla in the sea cucumber gut in our study), supporting a symbiotic interaction with their host [3, 103]. This phylum is composed of diverse physiological types that exist from strictly anaerobic bacteria like *Bacterodetes* sp. (present in the gut samples of this study), to facultative anaerobes such as *Lutibacter* sp. (a dominant genus found in *H. leucospilota*,), and strictly aerobic bacteria—*Flavobacteria* [17, 67, 103]. It has been increasingly recognized that members of *Bacteroidota*, are an integral part of their host metabolism due to their specialized capacity for degradation of high molecular weight organic matter such as protein and carbohydrates, as well as organic pollutants [73, 103, 123, 124]. In our study, such function (e.g., carbon degradation) was found significantly expressed in the gut of the sea cucumber, suggesting the beneficial and important existence of *Bacteroidota* members (e.g., *Flabacteriaceae* which was in high abundance compared to skin and environmental samples), for the breakdown complex molecules, as well as for biosynthesis activity (less expressed in the skin microbiome functional profile, [61]). Our results here support the hypothesis that the interplay between host-selective mechanisms and inherent host conditions modulate contrasting intra-host microbiome composition (skin and gut) in the sea cucumber *H. leucospilota*. It is important to highlight, however, that due to methodological limitations in our study (i.e., small sample size and lack of shotgun metagenomic data), these results should be interpreted with caution.

The Tolo Harbour, like many urbanized and industrialized estuaries around the globe, has been radically altered by historical and ongoing anthropogenic activities. Such alterations have impacted local biodiversity and the overall ecosystem functioning [15, 16, 34, 68]. At the organismal level, urbanization and pollution are known to influence the physiology, behaviour and life history of diverse marine animals [31, 79, 112]. These effects can also be observed in the complex assembly of animal-associated microbial communities and the functions they provide to their hosts [36, 89, 106, 110]. Alterations in host-associated microbiomes driven by urbanization and pollution are typically characterized by two types of outcomes: (1) host showing resilience due to the presence and enhancement of beneficial microbial members [35, 87] or (2) dysbiosis as a result of host dysregulation [55, 64]. Our study is likely to indicate the former, as we detected multiple dominant microbes with a beneficial role, such as *Rhodobacteriaceae* (keystone species in sea cucumber intestinal system, [118, 123, 124] and *Rhizobiaceae* (potentially aiding in pollutant breakdown, [100]) in sea cucumber However, further studies are needed to test this hypothesis, disentangling the microbial contribution to the host’s survival and tolerance to marine pollution.

## Conclusion

Marine nutrient pollution is an important driver modulating the structure and function of microbial communities. Spatial clines in the intensity and magnitude of this driver can result in different patterns of environmental filtering, even across short geographic scales. However, for microbial communities associated with marine animal hosts (e.g., the sea cucumber *H. leucospilota*), there are additional mechanisms influencing their composition and abundance. Such mechanisms are underpinned by intrinsic characteristics of their host (e.g., body compartments, biochemistry composition, immune systems), resulting in intra-individual differences in associated microbiomes, and their divergence from the environmental source. These findings support the hypothesis of an intrinsic capacity of the host to regulate its microbiome. Such regulation favours specific microbial functional pathways that may play an important role in the survival and physiology of the animal host, particularly in high polluted areas. Despite the observed differences in the environment and sea cucumber hosts, there was a small component of the microbial community (core microbiome) that was constant across the pollution cline and the animal body parts, suggesting that other mechanisms are also involved in the control of microbial communities in these animals.

## Supplementary Information

Supplementary material 1.

## Data Availability

DNA sequences used for this study are available at the NCBI Sequence Read Archive accession SRR14599054 to SRR14599073 and Bioproject PRJNA731335. All data and scripts can be downloaded from Dryad (https://doi.org/10.5061/dryad.qv9s4mwnk).
